# Clinical application of instant 3D printed cast versus polymer orthosis in the treatment of colles fracture: a randomized controlled trial

**DOI:** 10.1186/s12891-024-07212-8

**Published:** 2024-01-31

**Authors:** Ya-Ping Xiao, Hai-Jia Xu, Wen Liao, Zhang-Hua Li

**Affiliations:** The Department of Orthopedic Surgery, Wuhan Third Hospital, Tongren Hospital of Wuhan University, No. 241, Pengliuyang Road, Wuhan, 430000 Hubei Province P.R. China

**Keywords:** Colles fracture, 3D printing, Distal radius fractures, External fixation, Splint, Orthosis

## Abstract

**Background:**

The shortcomings of plaster in water resistance, air permeability, skin comfort, fixed stability and weight of wearing are still to be solved. 3D printed cast can overcome the above shortcomings. At present, there is a relative lack of data on the clinical application of 3D printed cast, probably due to its complexity, relatively long operating time, and high price. We aimed to compare and evaluate the short-term effectiveness, safety and advantages of 3D printed wrist cast versus polymer orthosis in the treatment of Colles fracture.

**Methods:**

Forty patients with Colles fracture in our hospital from June to December 2022 were selected and divided into an observation group (20 patients, treated with instant 3D printed cast) and a control group (20 cases, treated with polymer orthosis). Both groups treated with manual reduction and external fixation. The visual analogue scale (VAS), immobilization effectiveness and satisfaction scores, Disability of the Arm, Shoulder and Hand (DASH) score, complications and imaging data were collected and compared before immobilization and at 2, 6 and 12 weeks after the fracture.

**Results:**

VAS at 2 weeks after the fracture was significantly lower in the observation group than in the control group ( *P* < 0.05). The immobilization effectiveness and satisfaction scores at 6 weeks after the fracture were significantly higher in the observation group than in the control group (all *P* < 0.05). The DASH scores at 2 and 6 weeks after the fracture were significantly lower in the observation group than in the control group (all *P* < 0.05). There wasn’t rupture of the printed cast or orthosis in both groups. There were 2 cases of skin irritation in the control group, and no skin irritation occurred in the observation group. The palmar tilt angle and ulnar inclination angle at 2 weeks and 12 weeks after the fracture were significantly higher in the observation group than in the control group (all *P* < 0.05).

**Conclusions:**

Both instant 3D printed cast and polymer orthosis are effective in the treatment of Colles fracture. But instant 3D printed cast is better than polymer orthosis in areas of good clinical and imaging performance, and high patient satisfaction and comfort.

## Introduction

Fractures have been immobilized in a similar manner for centuries with little change [1]. Plaster or splint is a most widely used method for fracture immobilization in China [2]. The technique has changed little since it was invented in the 10th century. The shortcomings of plaster, such as water resistance, air permeability, skin comfort, fixed stability and weight of cast wear are still to be solved [3]. Recently polymer orthosis has replaced traditional plaster for the immobilization of fractured limbs and become the most commonly used external fixation material for fractures treatment in Chinese hospitals [4]. With the development of science and technology, 3D printing has become more common in the treatment of upper extremity fractures. 3D printed cast offers potentially excellent features that improve patient care and satisfaction [5].

3D printing technology is becoming more practical and has developed rapidly in the medical field [6]. 3D printing technology is widely used in orthopedics, including patient education, surgical training, and preoperative preparation [7]. It can plan patient-specific surgical guidelines and print customized splints and prostheses. 3D printed cast is personalized and perfectly suitable for patient anatomy and pathology, which has the advantages of light, breathable, washable, dirt-resistant and sand-proof characteristic and improves the comfort and satisfaction of patients [8, 9]. In addition, 3D printed cast can also be customized to avoid covering wounds and injured areas to prevent aggravation of injury or delay in treatment. At present, there is a relative lack of data on the clinical application of 3D printed cast, probably due to its complexity, relatively long operating time, and high price [8–11]. Instant 3D printing can quickly print the cast, which can be quickly used in the clinic practice, without long waiting, to meet the needs of fractured patients.

We hypothesized that instant 3D printed cast would have similar properties and functions to polymer orthosis. The aim of this study was to test the clinical feasibility and safety of personalized instant 3D printed cast, and to evaluate and compare the clinical outcomes and radiographic results between instant 3D printed cast and polymer orthosis for the immobilization of Colles fracture.

## Methods

### Selection criteria

The inclusion criteria were as follows: (1) Patients met the diagnostic criteria of Colles fracture. (2) Anteroposterior and lateral wrist radiographs were taken in the radiology department. (3) The type of the fracture was closed or grade I open Colles fracture. (4) Anatomical or functional reduction was achieved after manual reduction. (5) The time from injury to medical attention was ≤ 48 h. (6) Patients could successfully complete the follow-up. (7) Patients voluntarily accepted treatment with polymer orthosis or instant 3D printed cast.

Exclusion criteria were as follows: (1) Severe open fractures. (2) Severe systemic diseases, unable to cooperate with manual reduction; (3) Old or pathological fractures. (4) Manual reduction failed to achieve functional reduction. (5) Fractures associated with vascular or nerve injuries requiring surgical treatment.

### Study design

This study was a randomized controlled study without blindness. Both the investigators and the patients were aware of the treatment methods. This study was authorized by the Hospital Medical Ethics Committee of our Hospital. A total of 40 patients in the emergency department of our hospital from June to December 2022 were included in this study. Evaluation at the time of presentation in the emergency department included history taking, physical examination, and confirmation of the suspected injury on radiographs. Written informed consent to undergo external fixation treatment and be included in the study was obtained from all patients.

Patients who met the inclusion criteria of Colles fracture were randomly divided into two groups by random number table method. The manual reduction and immobilization of the two groups were performed by the same doctor. In the control group, 20 cases were treated with external fixation of polymer orthosis. In the observation group, 20 patients were treated with external fixation of instant 3D printed cast. There weren’t significant differences in baseline data between the two groups (all *P* > 0.05), as shown in Table 1, indicating comparability between the two groups.

**Table 1:** Comparison of baseline data between the two groups

Parameters	Observation group	Control group	t/χ^2^	*P*
Cases	20	20		
Gender(Cases)			0.417	0.519
Male	7	9		
Female	13	11		
Affected side(Cases)			0.107	0.744
Left	8	7		
Right	12	13		
Age(Years)	45.7 ± 16.2	44.2 ± 19.0	1.063	0.288
Time from injury to medical attention(Hours)	12.1 ± 12.2	11.9 ± 11.4	1.041	0.298

### Therapeutic methods

**Fig. 1:**
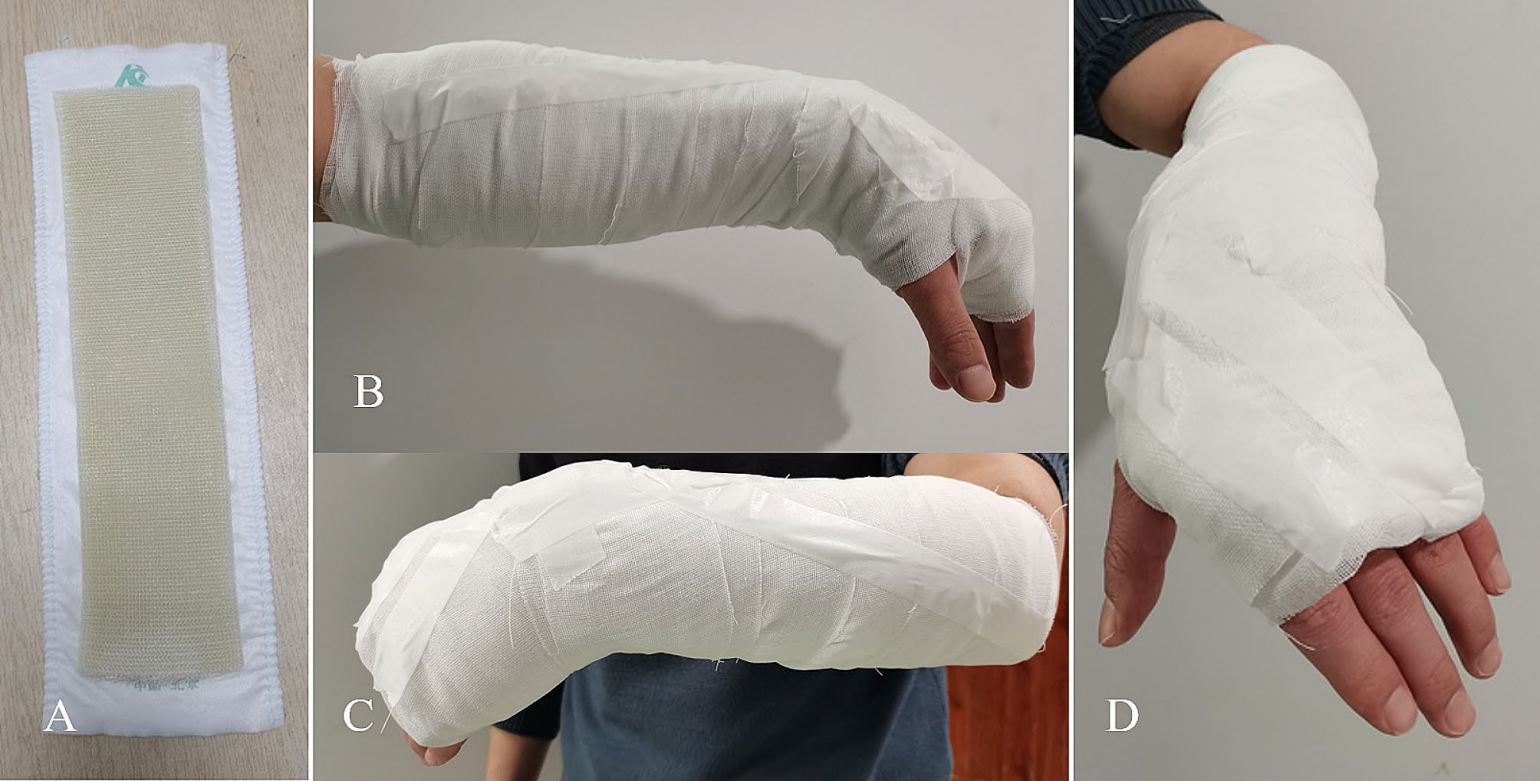
Treatment of Colles fracture patients with external fixation of a polymer orthosis. **A**: The white one in the picture is the pad and the polymer orthosis is on it. **B**: Medial visual field observation of polymer orthosis external fixation; **C**: The external fixation of the polymer orthosis was observed in the lateral visual field; **D**: Polymer orthosis external fixation in the anterior field of view

In the control group, the polymer orthosis was used for external fixation. The surgeon asked the patients to take off the clothes of the affected limb, and explained the treatment method and purpose to reduce the patient’s fear and obtain the patient’s cooperation. Fracture reduction was performed by manipulation according to the limb deformity and the X-ray images. After successful reduction, the assistant maintained the palmar-flexion angle and ulnar deviation angle. Finally, the surgeon performed external fixation with a polymer orthosis (Beijing Jinwei Kangda Medical Instrument LTD., Beijing, China) (Fig. 1).

**Fig. 2:**
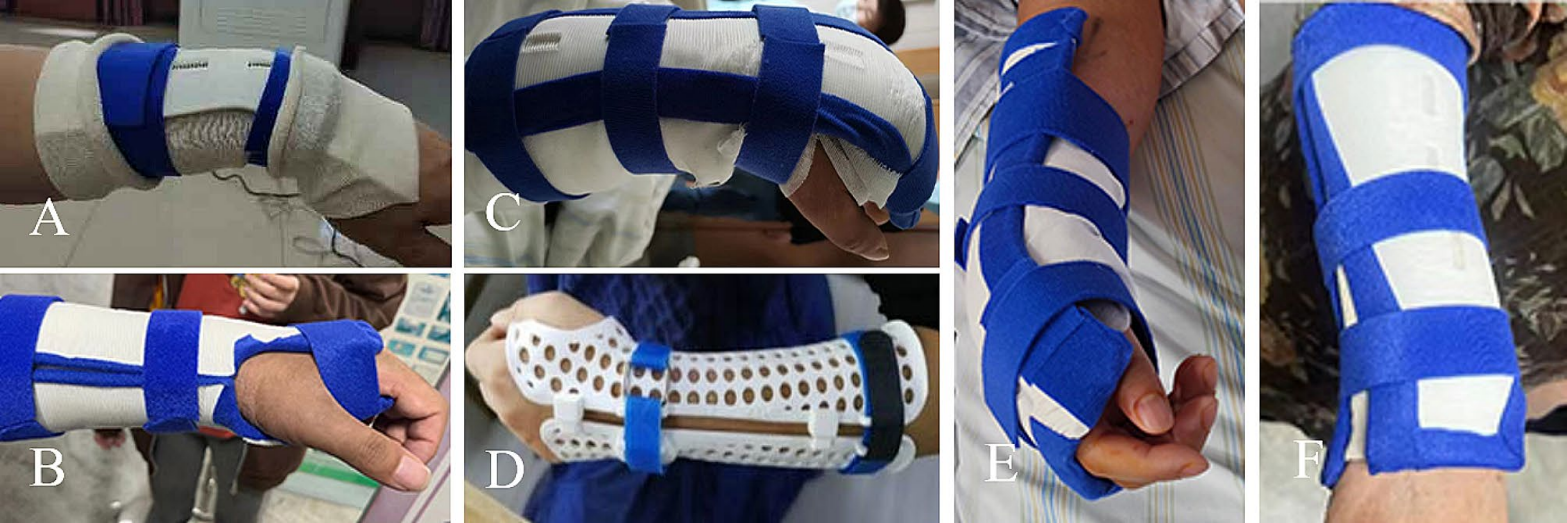
Photographs of the Colles fracture patients immobilized with the 3D printed casts. **A**: Case 1; **B**: Case 2; **C**: Case 3; **D**: Case 4; **E**: Case 5; **F**: Case 6

The observation group was fixed with 3D printed cast. The reduction method was the same as the above. After satisfactory fracture reduction, the patients were treated with external fixation of instant 3D printed cast (Fig. 2).

Anteroposterior and lateral wrist radiographs were performed weekly for the first two weeks in both groups to assess fracture alignment. If the local symptoms were aggravated and the blood supply was impaired, the patients were asked to see a doctor in time and adjust the brace. The malalignment caused by fracture displacement during fixation should be adjusted in time. More often than not, closed reduction again restores the fracture position. If closed reduction failed, open reduction and internal fixation were chosen. The external fixator was removed after 6–8 weeks according to the healing of the affected limb. All patients were asked to come to the outpatient clinic at the 2nd, 6th, and 12th week after injury for anteroposterior and lateral wrist radiographs and various scale evaluations (see the Outcome measures section below).

### The manufacturing process of instant 3D printed cast

#### Medical data collection

**Fig. 3:**
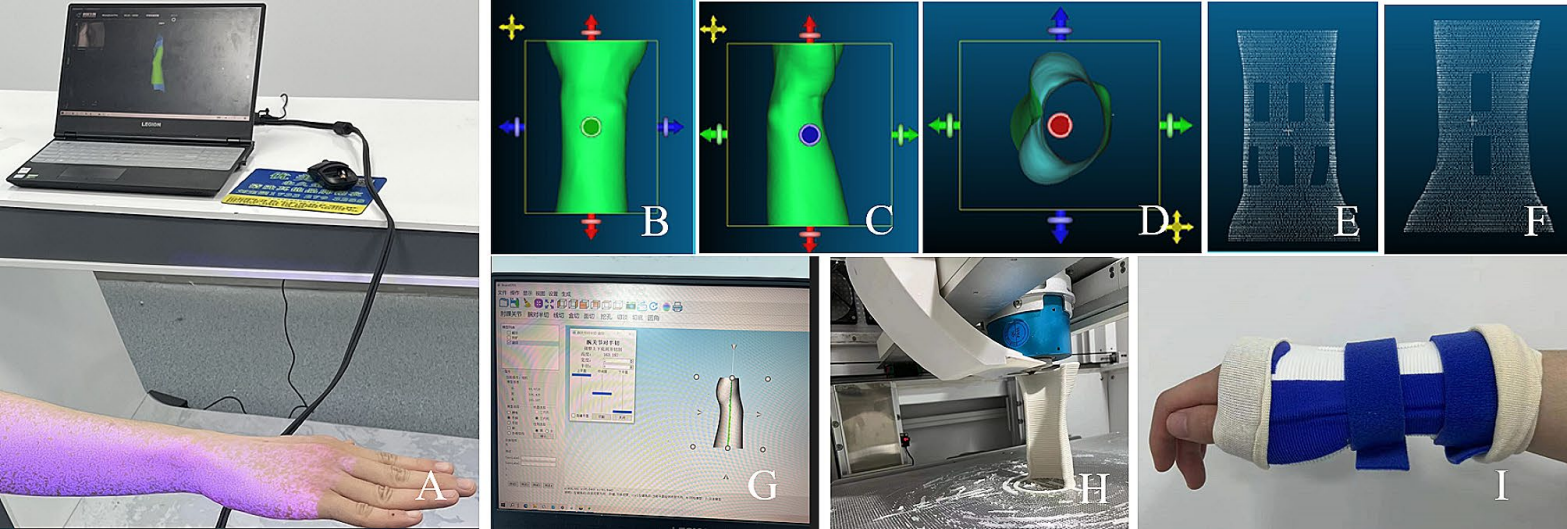
Manufacturing process of 3D printed cast. **A**: 3D scanning of the fracture site was performed using Einsan pro 2x handheld 3D scanner. **B**: The BY-3D-I instant printer slicing system was used to segment the model, and the anteroposterior view was obtained after segmentation. **C**: The segmented model of the lateral view; **D**: The segmented model of the axial view; **E**: The anteroposterior view of point cloud map automatically generated after cutting by the slicing system; **F**: The lateral view of point cloud map automatically generated after cutting by the slicing system; **G**: A 3D printed cast had been designed by a computer; **H**: A photo that the Instant 3D Cast Printer was printing a 3D printed cast; **I**: A photo of a patient with a Colles fracture wore a 3D printed cast

The Einsan pro 2x handheld 3D scanner (Wuhan Biyin Biotechnology Co., LTD., Wuhan, China) was used to scan the fracture site. The scanning distance should maintain in the appropriate area (Fig. 3A). Scanner parameters are shown in Table 2. The positioning of the scanned subject was placed in what the clinician considers to be the immobilized position. When the distance is appropriate, the system display will prompt green. The system will prompt “too close” or “too far” for improper distance. So the operator will adjust the distance in time. The operator should hold the scanner smoothly and scan at a uniform speed. Finally, the data obtained after scanning would be saved into STL format for output.

**Table 2:** Parameters of the Einsan pro 2x handheld 3D scanner

Parameters	
Light source form Scanning accuracy Volume accuracy Scanning & splicing speed Scanning mode Scanning depth of field Scanning range Space point distance Calibration method Standard working distance Color scanning Equipment size Equipment weight Transmission mode	White LED light source, visible light Maximum 0.05 mm 0.05 + 0.1 mm/m 1,200,000 points/second, 20FPS Mark splicing, feature splicing, texture splicing, hybrid splicing 200-700 mm ≤ 430 mm*450 mm 0.25-3 mm Fast calibration, accurate calibration 470 mm ± 30 mm Support ≤ 112 mm×114 mm×241 mm 703 g ± 5 g (excluding line) USB3.0

#### Optimization of 3D models

Scanning data were processed using a BY-3D-I instant printer slicing system (Wuhan Biying Biotechnology Co., LTD., Wuhan, China). First, the fracture site was extracted, and then the model was cut and segmented to obtain the area covered by the personalized external fixation cast (Fig. 3B-D). The system automatically thickened the model to leave a space for pad to cover the fracture site. The thickening size was determined according to the situation. The system automatically optimized the model surface and automatically generated the cast point cloud map (Fig. 3E, F).

#### Design of personalized external fixator

The wrist was selected as the center plane of the three-dimensional model after system optimization. The three-dimensional model was divided into two parts or a separate part of the orthosis on this plane. Then the edge of the wrist model was automatically drawn into the external contour of the personalized external fixation cast by the software. The designer could select the personalized ventilation hole pattern in the database according to the patient’s preferences and treatment requirements. The software would automatically generate the cast pattern (Fig. 3G).

#### 3D printing process

The overall size of the patient model needed to be increased by about 3 mm because the fracture site needed to be covered with pad before cast immobilization. The personalized external fixation cast was manufactured by fused deposition modeling (FDM) technology of 3D printing technology (Fig. 3H). BY-3D-I instant 3D external fixation printer (Wuhan Biying Biological Technology Co., LTD., Wuhan, China) served as the printer. The parameters of the 3D printer are shown in Table 3.The material was made of polyester fiber polymer material (Wuhan Biying Biotechnology Co., LTD., Wuhan, China). The wrist cast typically took approximately 10 min to print and can be quickly applied to clinical treatment (Fig. 3I). After processing, the support structure was removed and the surface of the model was simply polished to remove obvious burrs, so as to reduce the damage to the skin. Firstly, the fracture site was bandaged with a medical pad, and then the treated cast was installed and immobilized to the fracture site by several velcro straps.

**Table 3:** Parameters of the BY-3D-I instant 3D external fixation printer

Parameters	
Filament diameter Nozzle temperature Layer hight Nozzle speed Printing molding technology Molding accuracy Printing orthosis density Machine size	4-5 mm Above 200 degrees Celsius 0.5-1.5 mm 40 mm/s Melt deposition L < 100 mm: ±0.5 mm;L ≥ 100 mm: ±0.5% x L 0.74-0.81 g/cm^3^ 1250 mm×850 mm×1750 mm

The instant 3D external fixation printer used in this study is highly intelligent and the operation process is simplified, so that clinicians can quickly master the printing process and apply it to the patients. Clinicians can learn and become proficient in using the 3D printing process (scanner, software program, etc.) within a week. Wrist 3D printed cast can be printed in just 10 min. The scan takes about 5 min. It takes up to 5 min to operate computer system, and up to 5 min to process and put it on the patient after printing. Therefore, a wrist 3D printed cast takes about 25 min from the beginning of preparation to the patient wearing it.

#### Outcome measures

The visual analogue scale (VAS) before immobilization, 2 weeks and 6 weeks after the fracture was used to evaluate the wrist pain caused by Colles fracture. Immobilization effectiveness at 6th week after the fracture was assessed according to previous scoring scales, including stability of immobilization, blood circulation, wear-pressure-related pain, and pressure sores (Table 4) [10]. Patients’ comfort and satisfaction were evaluated at 6th week after the fracture by a satisfaction questionnaire, which includes questions related to treatment and assessment of patient satisfaction [12]. The Disability of the Arm, Shoulder, and Hand (DASH) scores at 2 and 6 weeks after the fracture were used to determine current functional ability [13]. Complications, such as orthosis or cast rupture, skin irritation or blister, fracture displacement and nonunion, were recorded. The wrist radiographs before immobilization, at 2 weeks and 12 weeks after the fracture were taken to measure the angle of palmar tilt and ulnar inclination angle of the injured wrist joint [14].

**Table 4:** Assessment of immobilization effectiveness of a orthosis [10]

Assessment Item	Assessment contents and grading standard			
	excellent-3	good-2	acceptable-1	poor-0
Stability of Immobilization	No loss of reduction	Slight shift but no need for re-manipulation	Reinforced same cast	Loss of reduction requiring further procedure
Blood circulation	Good terminal circulation with a florid complexion	Venous obstruction relief after physical movement or arm lifting	Pale skin, low temperature of the arm	Significant ischaemia of involved limb, compartment syndrome
Wear-pressure-related pain	No pain	Slight pain with a minor influence on sleep	Mild pain causedpoor-quality sleep	Severe pain caused difficulty falling asleep
Pressure sores	No abnormality of the skin	Non-blanchable erythema of the intact skin	Skin breakdown or bleeding blister	Full thickness skin loss

### Statistical analysis

The collected clinical data were processed and analyzed using IBM SPSS 19.0 statistical analysis software [15]. Measurement data were expressed as mean ± standard deviation. The *t* test was used for inter-group comparison. Analysis of variance was used for intra-group comparison. Counting data were compared by chi-square test. Nonparametric test was used to compare data with uneven variances. The level of significance was set at less than *P* 0.05.

## Results

All patients in both groups successfully completed 12 weeks follow-up (Table 5). The VAS in both groups were significantly lower at 2 and 6 weeks after the fracture than before immobilization (all *P* < 0.05). The VAS in both groups was significantly lower at 6 weeks after the fracture than at 2 weeks after the fracture (all *P* < 0.05). But the VAS at 2 weeks after fracture were significantly lower in the observation group than in the control group (An example shown in Fig. 4) (*P* < 0.05). The immobilization effectiveness score and immobilization satisfaction score at the 6th week after the fracture were significantly higher in the observation group than in the control group (all *P* < 0.05). The DASH scores at 2 and 6 weeks after the fracture were significantly lower in the observation group than in the control group (all *P* < 0.05). 3D printed cast or orthosis weren’t broken in the two groups. There were 2 cases of skin irritation in the control group, and no skin irritation occurred in the observation group. Complications such as skin blister, obvious fracture displacement and non-union weren’t reported in the both groups. The palmar tilt angle and ulnar inclination angle were significantly increased in the both groups at the 2nd and 12th week after the fracture compared with before immobilization (all *P* < 0.05). The palmar tilt angle and ulnar inclination angle in the both groups were decreased at the 12th week after the fracture compared with the 2nd week after the fracture (all *P* < 0.05). However, the palmar tilt angle and ulnar inclination angle at 2 and 12 weeks after fracture were significantly increased in the observation group compared with the control group (all *P* < 0.05).

**Table 5:** Comparison of clinical data between the observation group and the control group

Parameters	Observation group	Control group	*t*	*P*
VAS				
Before immobilization	7.2 ± 1.3	7.0 ± 1.4	1.333	0.184
At 2 weeks after fracture	1.6 ± 0.48*	1.8 ± 0.38*	-2.197	0.033
At 6 weeks after fracture	0.69 ± 0.32*^#^	0.74 ± 0.33*^#^	-0.316	0.755
Immobilization effectiveness score	9.8 ± 0.96	9.4 ± 1.07	3.849	0.000
Immobilization satisfaction score	11.6 ± 1.4	10.8 ± 1.8	4.686	0.000
DASH				
At 2 weeks after fracture	20.2 ± 5.7	22.6 ± 5.7	-5.781	0.000
At 6 weeks after fracture	8.1 ± 3.9^#^	10.2 ± 3.1^#^	-5.319	0.000
Palmar tilt angle				
Before immobilization	-5.7 ± 4.0	-5.7 ± 4.1	-0.380	0.704
At 2 weeks after fracture	11.1 ± 2.8*	10.0 ± 3.2*	3.628	0.000
At 12 weeks after fracture	9.8 ± 2.7*^#^	8.1 ± 2.7*^#^	5.604	0.000
Ulnar inclination angle				
Before immobilization	8.5 ± 3.6	8.8 ± 2.9	0.747	0.456
At 2 weeks after fracture	20.8 ± 2.5*	19.1 ± 2.8*	9.146	0.000
At 12 weeks after fracture	19.7 ± 2.5*^#^	17.5 ± 2.6*^#^	11.278	0.000

* Compared with before immobilization, *P* < 0.05

# compared with the 2nd week after fracture, *P* < 0.05

**Fig. 4:**
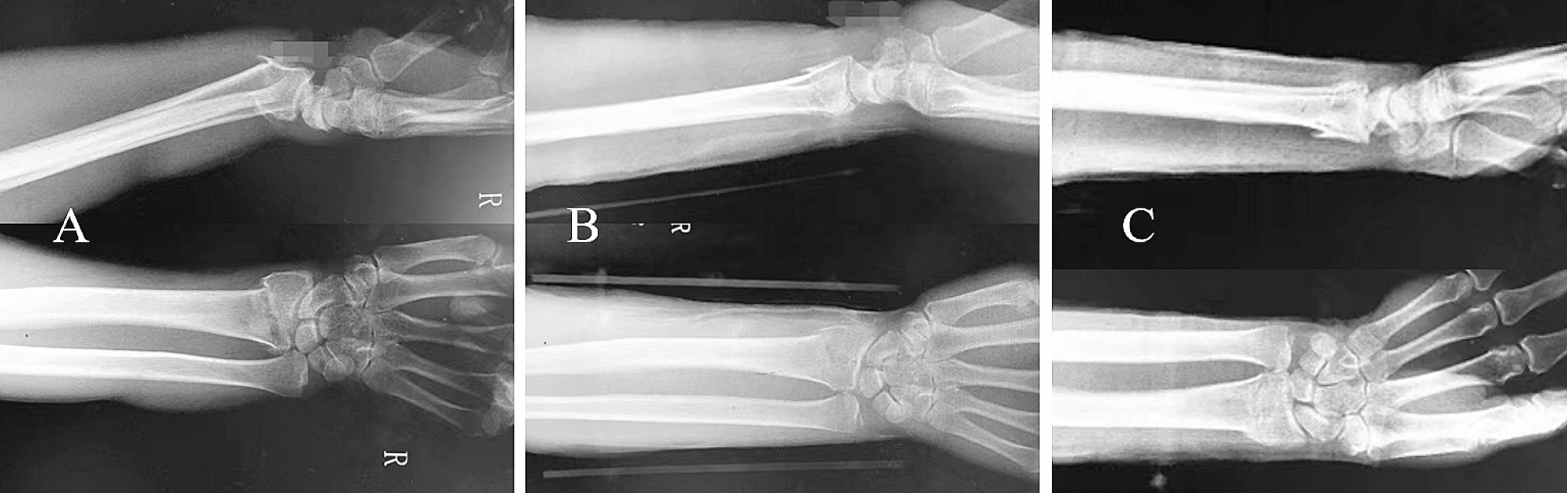
A 60-year-old female with Colles fracture was treated with a polymer orthosis. **A**: Before treatment; **B**: After immobilization; **C**: After removing the immobilization

## Discussion

Although traditional fixation techniques such as casts and splints have been widely used for external fixation of fractures, few modifications have been made to them [5, 12]. In recent years, polymer orthosis has become more and more popular in China [4]. Although traditional plaster and polymer orthosis are highly favored from a clinical perspective due to their low cost, strength, and ease of application, the disadvantages of traditional splints are also obvious such as heavy weight, low breathability, inability to get wet or clean, and lack of transparency [16–18]. Compared to traditional plaster, a 3D-printed cast can be tailored to fit the patient’s anatomy and pathology, thus increasing the patient comfort and satisfaction [8, 9]. Personalized 3D printed cast increases direct skin visualization to minimize the need of keeping dry to maintain comfort [8]. At present, Use of 3D printed technology is showing promise in orthopedic clinical practice.

Hoogervorst et al. [19] showed in the cadaver model that, in immobilizing subacute distal radius fractures, compared with traditional fiberglass plaster, 3D printed cast had non-inferior performance and had clinical application value. Chen et al. [10] conducted a clinical trial using a 3D printed cast for forearm fracture and concluded that it increased patient comfort and satisfaction, but their study included only 10 patients ranging in age from 5 to 78 years. Keller et al. [9] published a multidisciplinary workflow study to verify the feasibility of mass production of patient-specific 3D printed devices for hand and wrist rehabilitation in hospitals. There aren’t clinical studies that have examined the efficacy of instant 3D printed cast for the management of distal radius fractures.

We designed this study under the assumption that the properties and functions of instant 3D printed cast is similar to those of polymer orthosis. We hypothesized that the instant 3D printed cast would be effective in good clinical and radiographic findings and high patient satisfaction and comfort. Our study confirmed our hypothesis. Both the instant 3D printed cast and the polymer orthosis could effectively relieve the pain of Colles fracture. The pain was gradually relieved as the fracture healed, but the instant 3D printed cast could relieve the pain more effectively at 2 weeks after fracture than that of the polymer orthosis (*P* < 0.05). According to the questionnaire score, the immobilization effectiveness score and immobilization satisfaction score were significantly higher in instant 3D printed cast group than in the polymer orthosis group. The DASH score was significantly lower in instant 3D printed cast group than in the polymer orthosis group. All these factors were beneficial to pain relief and dysfunction improvement. After 3 months of follow-up, the angle of palmar tilt and ulnar inclination angle in both groups increased significantly at 2 weeks and 12 weeks after fracture compared with before fracture. As the fracture healing, the angle of palmar tilt and ulnar inclination angle in both group decreased at 12 weeks after the fracture compared with 2 weeks after the fracture. But the angle of palmar tilt and ulnar inclination angle were significantly higher in the observation group than in the control group. We confirmed that both instant 3D printed cast and polymer orthosis can effectively treat Colles fractures, but instant 3D printed cast is superior to polymer orthosis.

At present, 3D printed cast has been widely used to construct high-precision bone models, which can be used for advanced imaging and preoperative simulation in preoperative planning [20, 21]. 3D printing technicians have mastered the professional knowledge and skills of wrist therapists to accurately scan the patient’s upper limbs. The use of 3D printing equipment can be used to manufacture customized 3D printing braces to improve clinical practice for the field of orthopedic rehabilitation treatment. Because the technology is still in its infancy, the actual cost of producing 3D printed brace is in flux. Although there are fixed costs associated with purchasing a scanner and printer, the printing materials are relatively inexpensive. The fixed costs of 3D printing are expected to decrease over time.

The goal of manufacturing 3D printed orthosis, with waterproof, washable, lightweight, static, and removable padded materials, is to improve the quality of life of patients and their compliance of immobilization methods [8, 9]. The quality of these orthosis has a huge impact on the wearer’s experience, especially for children, the elderly, athletes who need regular skin observation [8, 10, 11]. However, the orthosis must be safe and functionally noninferior to the conventional brace currently available. In terms of the wearable characteristics of the orthosis, the instant 3D printed cast group was significantly better than the polymer orthosis group, probably due to the more streamlined design and lighter structure of the instant 3D printed cast than the polymer orthosis. The function, effectiveness, comfort and satisfaction of the observation group were better than those of the control group. Two patients in the control group needed to replace the orthosis again because of discomfort. There was no case of discomfort in the observation group. These are consistent with our hypothesis and with previous studies [11].

Polymer orthosis is widely used in fracture treatment in hospital emergency department and orthopedic clinical practice [5]. From the perspective of material properties, polymer orthosis is light and easy to shape, which is better than traditional plaster. These features are the key to its wide use. Polymer orthosis is widely favored in clinical practice because of their low cost, high strength and ease of application. However, parents of children who wear polymer orthosis do not always satisfy and need further improvement. Key concerns for parents and children include heavy weight, reduced movement of the affected limb due to fear of sweating or getting wet, reduced movement of the affected limb to perform rehabilitation functions, and inability to remove or see the skin under the orthosis to adequately examine for breakdown, ulcers, or pressure sores [5, 22]. The 3D printed cast can open windows and openings at the affected site, even allow the cast to be removed for skin examination if necessary.

While fit and safety are ensured, cost and printing time are two significant factors in the clinical determination of treatment regimens with 3D printing technology. Presumably, the advantages of 3D printed cast far outweigh all the advantages of polymer orthosis, thermoplastic orthoses, or prefabricated orthoses. The advantages of 3D printed cast include but not limited to high fit, aesthetic appeal, lightweight structure, waterproof design, and improved medical rehabilitation and skin care capabilities. However, its cost is higher than these of traditional brace, which increases the economic burden of patients. This is an important factor to consider for health care professionals considering 3D printed cast. In clinical practice, the cost of 3D printing scanner and printer is the main cost for clinical use, while the printing materials are relatively inexpensive. The 3D printing equipment usage fee mainly increases the cost of patients. As the scale of clinical application of 3D printing cast expands, the costs of 3D printing cast are expected to significantly decrease over time. 3D printed cast was functionally noninferior to traditional brace, while providing a water-resistant, lightweight, and breathable alternative [11]. Except for mild irritation, there weren’t adverse reactions in the short term. Instant 3D printing takes about 10 min to print the wrist brace. After completion of printing, it can be applied to patients immediately after product improvement, which does not require patients to wait too long, and can quickly meet clinical needs. This is one of the advantages of instant 3D printing braces and the key to their potential for widespread clinical use.

The emerging technologies allow clinician to use better casts and braces, providing more precise fitting for reliable, seamless, and waterproof immobilization. The successful application of this technique has a huge impact on injury care and patient satisfaction, especially in the field of orthopedics. For patients who may require brace treatment, such as orthopedic injuries and chronic diseases (such as arthritis and joint deformities), 3D printed braces have great potential in improving the living quality of patients.

The limitations of this study are as follows. Medical conditions were not managed, and underlying medical conditions may have affected the results of this functional wear and safety study. Since 3D printing technology is a new technology, patients have a “cool factor” and may be subjectively biased toward 3D printing braces. Data bias may have occurred when data were collected. Further studies need to blind volunteers to prevent subjective bias. The wearing time in this study was short, and long-term wearing may cause more complications in patients with fragile skin. Future studies are needed to further evaluate the safety and benefits of 3D-printed casts in patients with orthopedic disease or injury. This study is a single-center study with a small sample size. Further well-designed, randomized, controlled polycentric trials are needed to clarify the clinical application of this new device.

## Conclusions

Both instant 3D printed cast and polymer orthosis are effective in the treatment of Colles fracture, But instant 3D printed cast is better than polymer orthosis in areas of good clinical and imaging performance, and high patient satisfaction and comfort. This study is a preliminary clinical exploration, and further research is needed to verify our results, especially a large sample size multicenter controlled study.

## Data Availability

The datasets used and/or analysed during the current study are available from the corresponding author on reasonable request.
